# Title of the study: perceived social support and anxiety symptoms among the Palestinian pregnant women: a cross-sectional study

**DOI:** 10.1186/s12884-025-08111-z

**Published:** 2025-09-30

**Authors:** Maha Sudki Hmeidan Nahal, Sireen Ibrahim Bshareya, Ibtesam Medhat Mohamad Dwekat, Khaled W. Nahal

**Affiliations:** 1 MSN Maternal &Child Health, PhD in health care sciences. Director of Midwifery Department, Faculty of Health Professions, Al-Quds University, Abu Dis Campus, Jerusalem, Palestine; 2 Community Mental Health/ Psychotherapy, Al-Quds University, Abu Dis Campus, Jerusalem, Palestine; 3 MSN Maternal &Child Health, PhD in Community Health Development. Faculty of Health Professions, Al Quds University, Abu Dis Campus, Jerusalem, Palestine; 4 Faculty of Medicine, ARAB AMERICAN UNIVERSITY, Jenin, Palestine

**Keywords:** Perceived social support, Pregnancy-related anxiety, Anxiety symptoms

## Abstract

**Background:**

Anxiety during pregnancy is a common concern that can significantly affect maternal mental health. The ongoing political instability in Palestine contributes to increased anxiety among pregnant women, with perceived social support playing a key moderating role. This study aims to explore the relationship between perceived social support and anxiety symptoms among pregnant women in the West Bank, Palestine.

**Methods:**

A descriptive cross-sectional study was conducted among 305 pregnant women recruited from four major hospitals in the West Bank, Palestine. Data were gathered using three reliable questionnaires: the Medical Outcomes Study Social Support Scale (MOS-SSS), the Pregnancy-Related Anxiety Questionnaire-Revised 2 (PRAQ-R2), and the State Anxiety Inventory (SAI).

**Results:**

Participants reported high levels of perceived social support, with 265 (86.9%) reporting high emotional support, 245 (80.3%) high informational support, and 261 (85.6%) high tangible support. Moderate levels of anxiety symptoms were observed across both measures. The mean score on the PRAQ-R2 was 2.83 ± 0.5, and the mean SAI score was 50. Employment status was significantly and positively associated with anxiety symptoms on both the PRAQ-R2 (*p* = 0.015) and the SAI (*p* = 0.005). A moderate negative correlation was found between the number of pregnancies (*r* = -0.142, *p* = 0.013) and the number of children (*r* = -0.153, *p* = 0.007) and PRAQ-R2 scores. Additionally, a moderate inverse correlation was observed between MOS-SSS and SAI scores (*r* = -0.402, *p* < 0.001), while a weak inverse correlation was noted between MOS-SSS and PRAQ-R2 scores (*r* = -0.024, *p* = 0.672). Furthermore, a moderate positive correlation was found between overall SAI and PRAQ-R2 scores (*r* = 0.379, *p* < 0.001).

**Conclusion:**

In this study, pregnant women reported moderate levels of anxiety, despite experiencing high levels of perceived social support. Unemployed women and those with more children and pregnancies demonstrated lower levels of anxiety symptoms. This research highlights the importance of regular screening for prenatal anxiety and emphasizes the need to enhance social support systems to promote maternal mental health. Further research is needed to explore these relationships in greater depth.

## Background

Pregnancy-related anxiety is a significant public health concern, as it increases the risk of maternal mental health disorders and can negatively impact both the mother and the developing fetus [1]. Effective assessment and management are essential for helping women navigate the physical, psychological, and emotional changes that occur during pregnancy [2]. Common physical symptoms—such as morning sickness, fatigue, frequent urination, and breast tenderness—are often accompanied by ongoing worries about pregnancy and childbirth outcomes, which may worsen anxiety symptoms [3, 4]. Antenatal anxiety commonly manifests as persistent nervousness, insomnia, palpitations, teeth clenching, and muscle tension [5]. These symptoms can greatly affect a pregnant woman’s overall well-being and may hinder her ability to bond with her baby.

Several factors contribute to antenatal anxiety, including concerns about the baby’s health, unplanned pregnancies, and complications such as pre-eclampsia, instrumental deliveries, or miscarriage [4, 6]. Sociodemographic factors, including gender, exposure to environmental stressors, and the presence of underlying medical conditions, have also been identified as potential contributors to antenatal anxiety [7].

Global studies indicate that the prevalence rates of antenatal anxiety vary significantly, ranging from 15.6 to 49% [8]. However, there is a lack of research focusing specifically on the psychological effects of pregnancy within the Palestinian population, despite some studies suggesting even higher rates of anxiety. Reports indicate that antenatal anxiety in Palestine is a pressing public health concern, with a prevalence rate of 60.1% [9, 10].

Research among Palestinian university students and youth has revealed a high prevalence of anxiety and depression [11–13]. Gender differences have been noted, with female students experiencing more severe anxiety symptoms than their male counterparts [11, 12]. One of these studies, conducted during the COVID-19 pandemic, found that the severity of anxiety among Palestinian undergraduates varied significantly based on factors such as gender, year of study, sleep duration, smoking habits, source of food, and place of residence, rather than their field of study [11]. Furthermore, various studies underscore the specific challenges faced in Palestine, where ongoing political conflict, violence, restrictions on movement, limited coping resources, and a fragmented healthcare system contribute to increased stress and depression among young people, including pregnant women [11–14].

Pregnant women play a crucial role in family formation and shaping societal outcomes [15]. Therefore, prioritizing their mental health—especially regarding antenatal anxiety—in conflict-affected regions like Palestine is vital. While antenatal anxiety is a complex issue that can lead to depression, strong social support can serve as a protective factor by alleviating stress and reducing the risk of both anxiety and depression during pregnancy [16–18]. Thus, implementing anxiety screening, ensuring early detection, providing appropriate interventions, and offering timely support are critical steps for protecting the emotional health of pregnant women [19]. This focus not only supports mothers’ well-being but also enhances community resilience and fosters healthier future generations. We must emphasize the importance of tailoring targeted mental health interventions to the unique experiences of the Palestinian population.

However, studies examining anxiety symptoms in pregnant women within the Palestinian context remain limited, and the relationship between social support and anxiety is not well understood. Addressing the prevalence and severity of anxiety symptoms, as well as the level of perceived social support among pregnant women in Palestine, is crucial for improving maternal mental health and pregnancy outcomes.

## Materials and methods

### Design, setting, and sample

A cross-sectional study was conducted in four major hospitals, including two private and two governmental facilities, in the Ramallah Governorate of the West Bank, Palestine. The inclusion criteria consisted of pregnant women of any gestational age, aged 18 to 45 years, who attended antenatal clinics during the study period. This demographic is crucial for understanding maternal and fetal health. By focusing on a diverse range of gestational ages, researchers aim to gather comprehensive data on anxiety during pregnancy. Women with high-risk pregnancies or those with physical or mental health comorbidities were excluded from the study. This exclusion was necessary because such conditions could significantly confound the interpretation of the results and potentially elevate anxiety levels.

The total number of pregnant women attending the antenatal clinics at the selected hospitals each month was approximately 4,185. The required sample size was calculated using a single-proportion formula with a 95% confidence interval and a 5% margin of error, yielding a target of 352 participants. A proportionate quota sampling approach was first used to distribute the sample across the four hospitals, based on their monthly antenatal clinic referral rates. Within each hospital, eligible participants were then selected using a non-probability convenience sampling method. This approach resulted in a final sample of 305 pregnant women.

### Instruments

A self-reported questionnaire was used to evaluate the relationship between perceived social support during the antenatal period and the level of anxiety symptoms among Palestinian pregnant women. The study utilized three standardized tools: the Medical Outcomes Study Social Support Scale (MOS-SSS), the Pregnancy-Related Anxiety Questionnaire-Revised 2 (PRAQ-R2), and the State Anxiety Inventory (SAI). The baseline questionnaire also collected demographic and obstetric information, including age, gestational age, number of previous pregnancies, educational level, occupational status, place of residence, and family structure. The MOS-SSS was originally developed in English as a 19-item scale designed to assess social support among patients with chronic conditions [20]. Since its inception, it has been translated, adapted, validated, and utilized in several languages, including Portuguese [21], Chinese [22], and Arabic [23].

This study used the Arabic version of the MOS-SSS, which was validated in northern Jordan and modified to include 18 items by Hijazi et al. [23]. The Arabic version of the MOS-SSS demonstrates high consistency and excellent reliability, as indicated by a Cronbach’s α of 0.91. A recent systematic review has confirmed its potential for practical application in future studies [24]. Although it has not been specifically validated in Palestine, it has been deemed suitable for use among Bedouin Arab undergraduates living in Israel [25]. Additionally, confirmatory factor analysis has validated it within other Arab populations in neighboring regions, including Jordan [26] and Sudan [27]. These findings suggest that the instrument is suitable for assessing social support among Arabic-speaking populations, such as Palestinians.

The scale is categorized into three domains. Emotional support includes six items that reflect feelings of being cared for, receiving advice, sharing worries, having someone to listen, understanding problems, and experiencing love and affection. Informational support consists of four items that assess the availability of advice, guidance, and reassurance from healthcare professionals. Tangible support comprises eight items that measure the availability of practical assistance, such as help with daily tasks, spending time together, participating in activities, and receiving support during illness or periods of confinement.

The MOS-SSS was rated on a five-point Likert-type scale, ranging from 1 (“None of the time”) to 5 (“All of the time”). In this study, responses to “None of the time” and “A little of the time” were classified as low social support, “Some of the time” as moderate, and “Most of the time” and “All of the time” as high social support. Additionally, the mean score was transformed to a 100-point scale. Mean scores below 60% indicate low levels of social support, ratings between 60% and 80% reflect moderate social support, and scores above 80% indicate high levels of social support [27, 28].

The original Pregnancy-Related Anxiety Questionnaire (PRAQ) was developed by Van den Bergh and consisted of 34 items designed to assess anxiety symptoms in nulliparous women [29]. Following its initial development, the PRAQ underwent analysis to confirm that it specifically targeted pregnancy-related anxiety rather than general anxiety within the same demographic [30]. This analysis led to the creation of a shorter, 10-item version of the tool, which focuses on three key areas: fear of childbirth (3 items), fear of having a child with physical or mental disabilities (4 items), and concerns regarding one’s appearance during pregnancy (3 items).

In subsequent research, the tool was revised to ensure its applicability for both nulliparous and multiparous women, and this update was validated [31]. The revised tool, known as the PRAQ-R2, demonstrated high validity and reliability, with a Cronbach’s alpha exceeding 0.8. The PRAQ-R2 exhibited strong psychometric properties and has been validated in various cultural contexts [32–37], which supports its reliability and cultural sensitivity in assessing pregnancy-related anxiety among all pregnant women. Although this tool has not yet been utilized in Palestine, it has been applied in other Arab countries, including Saudi Arabia, Qatar, and Egypt, which further reinforces its validity for use in Palestine [38–40]. In this study, permission was obtained from the original authors to use the Arabic version of the PRAQ-R2 [38, 39].

This version employs a five-point Likert scale, ranging from 1 (“Never”) to 5 (“Always”), with total scores calculated by summing responses to all 10 items, resulting in a possible range of 10 to 50. Higher scores indicate a greater likelihood of experiencing pregnancy-related anxiety. A total score of less than 26 (mean ≤ 2.6) is considered low; a score between 26 and 34 (mean 2.61–3.4) is considered moderate; and a score above 34 (mean 3.41–5) is considered high.

We utilized the State Anxiety Inventory (SAI), developed by Spielberger et al. [41] in 1970. The SAI consists of 20 items, which were initially created in English and later translated into Arabic in an Egyptian study [42] for use in subsequent research within the Arabic population [43]. To our knowledge, the Arabic version has been previously used in the Palestinian context to assess anxiety levels among women in the West Bank during the COVID-19 pandemic [44]. Studies evaluating the reliability of the SAI have consistently demonstrated its validity and strong internal consistency across various research efforts [45, 46]. For this study, we employed the validated Arabic version of the SAI after obtaining permission from the authors in Egypt [42]. The scale uses a four-point Likert format, ranging from 1 (“Never”) to 4 (“Always”), with total scores ranging from 20 to 80. According to prior research, higher scores indicate greater levels of state anxiety. Based on previous studies [47], anxiety levels measured by the SAI scores were categorized into four groups: no anxiety (score = 20), mild anxiety [21–39], moderate anxiety [40–59], and severe anxiety (60–80).

Before data analysis, responses to 10 specific items (questions 1, 2, 5, 8, 10, 11, 15, 16, 19, and 20) were reverse-coded from a scale of 4 to 1. Furthermore, pilot testing was conducted with 10 pregnant women to evaluate the clarity, relevance, feasibility, and completion time of the questionnaire.

### Data collection

Data collection occurred between December 20, 2022, and February 10, 2023. During this timeframe, the researcher visited the hospital five days a week to recruit participants and gather data using a convenience sampling method. Official approval to conduct the study in governmental hospitals was obtained from the Palestinian Ministry of Health (MOH), while access to private hospitals was coordinated with their respective administrators. Eligible women who expressed a willingness to participate were approached by the researcher and informed about the study’s purpose, potential benefits, and the voluntary nature of their involvement. Participants completed a self-administered, paper-based questionnaire in a private setting within the clinic. The researcher was available to provide clarification as needed. Completing the questionnaire took approximately 20 to 25 min.

### Ethical considerations

This study was conducted in accordance with the ethical principles outlined in the Declaration of Helsinki [48]. Ethical approval was granted by the Ethics Committee at Al-Quds University (Ref. No. 262/REC/2022). Informed consent was obtained from all participants before data collection, with the consent form attached to the questionnaire. To ensure the emotional well-being of participants, a mental health specialist was available for support if needed, as the questionnaire addressed sensitive topics that could potentially cause emotional distress.

### Statistical analysis

The researchers conducted data entry and subsequently double-checked for outliers, missing data, and errors. The analysis was performed using version 27 of the Statistical Package for Social Sciences (SPSS). This study utilized both descriptive and inferential statistics. Descriptive statistics involved looking at how often certain scores appeared, figuring out percentages, average scores, and Standard Deviation (SD) for both the independent and dependent variables related to the MOS-SSS, PRAQ-R2, and SAI scales.

For the analysis of the SAI scale questions, researchers inversely coded items 1, 2, 5, 8, 10, 15, 16, 17, 19, and 20, Changing their values from 4 to 1. Inferential statistical analysis commenced with testing the normality of variable distribution using the Shapiro-Wilk and Kolmogorov-Smirnov tests. Since the data did not follow a normal distribution, researchers used nonparametric tests like the Mann-Whitney U test, Kruskal-Wallis H test, and Spearman’s correlation to look at differences and connections between the variables. To find out what influences mothers’ outcomes, a linear regression analysis was done using important independent factors along with the PRAQ-R2 and SAI scores. Predictors for the MOS-SSS were not analyzed, as no significant relationships were found.

## Results

### Participants characteristics

A total of 305 pregnant women participated in the study. Ages ranged from 19 to 40 years, and the mean age was 28 years (SD = 4.8). The majority of participants, 210 (68.9%), had completed university education, while 95 (31.1%) had a school-level education. Employment status revealed that 231 (75.7%) were unemployed, whereas 74 (24.3%) were employed. Based on gestational age, 48 (15.7%) were in their first trimester (1–13 weeks), 105 (34.4%) were in their second trimester (14–27 weeks), and 152 (49.8%) were in their third trimester (28–40 weeks). The basic characteristics of the participants are summarized in Table 1.

**Table 1:** Socio-demographic characteristics of the participating pregnant women (*N* = 305)

Variable	Categories	Frequency (%)	mean ± (SD)
Age	>30years	116 (70.8%)	28 ± (4.8)
	31–35 years old	68 (22.3%)	
	36–40 years old	21 (6.9%)	
Educational level	High school	95 (31.1%)	--------
	University degree	210 (68.9%)	
Occupational status	Employed	74(24.3%0	----------
	Unemployed	231(75.7%)	
Residency	City	90(29.5%)	-----
	Town	130 (42.7%)	
	Village	44 (14.4%)	
	Camp	41(13.4%)	
Gestational age (weeks)	1–13 weeks.	53(17.4%)	26 ± (10.67)
	14–27 weeks.	101(33.1%)	
	28–40 weeks	151(49.5%)	
Number of lived children	None	92 (30.19%)	1.68 ± (1.55)
	One child	60 (19.7%)	
	Two children	64 (21.0%)	
	Three children	51 (16.7%)	
	More than three	38 (12.5%)	
Did you have previous abortions or stillbirths	Yes	91(29.8%)	
	No	214(70.2%)	
Is the current pregnancy planned?	Yes	193(63.3%)	
	No	112(36.7%)	
Monthly income	< 2000 NIS	5(1.6%)	3984 ± (1804)
	2000–3499 NIS	129(42.3%)	
	3500–4999 NIS	93(30.5%)	
	> 5000 NIS	78(25.6%)	

*SD * Standard deviation, *NIS * New Israeli Shekel (currency)

### The Medical Outcome Study Social Support Scale (MOS-SSS)

Table 2 displays the frequency, percentage, mean, and standard deviation (SD) of perceived social support as measured by the Medical Outcome Study Social Support Scale (MOS-SSS). The majority of participating mothers reported high levels of social support across three domains: emotional support (265, 86.9%), informational support (245, 80.4%), and tangible support (261, 85.6%). The overall mean score was 84 (SD = 5.5), reflecting a generally high level of perceived social support among the participants.

**Table 2:** Results of the medical outcomes study social support scale (MOS-SSS) among the participating mothers (*N* = 305)

Domains of MOS-SSS scale	Level of support	Frequency (%)	Mean ±(SD)
Emotional support	Low	4 (1.3%)	86.7 **±** (6.4)
	Moderate	36 (11.8%)	
	High	265(86.9%)	
Informative support	Low	5 (1.64%)	80.9 **±** (6.2)
	Moderate	55(18%)	
	High	245(80.33%)	
Tangible support	Low	(2.3%)	84.6**±** (7)
	Moderate	37(12.13%)	
	High	261(85.6%)	
Total Mean of MOS-SSS scale			84 **±** (5.5)

### The Pregnancy-Related anxiety Questionnaire-Revised 2 (PRAQ-R2)

Table 3 shows that the average score for the Pregnancy-Related Anxiety Questionnaire-Revised 2 (PRAQ-R2) is 2.83 (± 0.5), which means that participants feel a moderate amount of anxiety. The highest mean score, 3.6 (± 0.8), reflects the fears pregnant women have about giving birth, while the mean score for concerns about their appearance is 2.6 (± 0.4). In contrast, the lowest mean score of 2.3 (± 0.3) relates to worries about their child’s well-being. In detail, 136 participants (44.6%) reported often experiencing delivery anxiety, and 97 (31.8%) indicated that they always feel nervous. Concerns about pain during labor and contractions were also common; 105 participants (34.4%) consistently expressed anxiety, while 133 participants (43.6%) frequently worried about these issues. However, only 67 participants (22.0%) reported frequently worrying about losing control during labor and fearing that they might scream.

**Table 3:** Results of the self-reported Pregnancy-Related anxiety Questionnaire-Revised 2 (PRAQ-R2) among the participating mothers(*N* = 305)

PRAQ-R2	Anxiety level	Mean ±(SD)	Range	The sum of the scores
Fear of giving birth	High	3.6 **±** (0.8)	3–15	36/50
Worries about physical appearance	Moderate	2.6**±** (0.4)	4–20	26/50
Worries about the child’s well-being	Low	2.3 **±** (0.3)	3–15	23/50
Overall score	Moderate	2.83 **±** (0.5)	10–50	28.3/50

Low anxiety level (Mean ≤ 2.6), Moderate anxiety level (Mean = 2.61–3.4) High anxiety level (Mean = 3.41 −5)

*SD* S standard deviation

### The state anxiety inventory scale (SAI)

The data revealed that the majority of mothers, 139 (45.6%), experience a moderate level of anxiety. Additionally, 125 (41%) report mild anxiety, 30 (9.8%) indicate no anxiety, and only 11 (3.6%) experience severe anxiety. The overall mean score for the 20 items on the State Anxiety Inventory (SAI) was 2.5 (SD = 0.46), corresponding to a total score of 50, which reflects moderate anxiety symptoms. These findings are presented in Table 4.

**Table 4:** Results of the self-reported anxiety level on the state anxiety inventory (SAI)among the participating mothers(*N* = 305)

Categories SAI	Frequency	Percentage (%)
No Anxiety < 20	30	9.8%
Mild State Anxiety (21–39)	125	41%
Moderate State Anxiety (40–59)	139	45.6%
Severe State Anxiety (60–80)	11	3.6%

*SAI * State anxiety inventor

### Correlations

The results of the current study indicated no significant correlations between mothers’ age, gestational age, number of pregnancies, number of children, monthly income, education, occupation, residency, number of abortions, planning of pregnancy, and the perceived social support scales and subscales (*p*-value > 0.05).

A significant difference was noted in maternal employment status concerning PRAQ-R2 and SAI scores, with unemployed mothers showing more favorable outcomes. Specifically, mothers with lower PRAQ-R2 and SAI scores were more likely to be unemployed (*p* = 0.015 and *p* = 0.005, respectively). A moderate negative correlation was observed between the number of pregnancies (*r* = −0.142, p-value = 0.013), the number of children (*r* = −0.153, p-value = 0.007), and the PRAQ-R2 scores. This finding suggests that symptoms of pregnancy-related anxiety significantly decrease among multiparous mothers with more children.

No significant correlations were found between pregnancy-related anxiety and mothers’ age, gestational age, or monthly income. Furthermore, state anxiety did not show significant associations with any of the mothers’ numerical sociodemographic factors (*p* > 0.05).

Table 5 showed a moderate negative relationship between the overall SAI score and the MOS-SSS score (*r* = −0.402, *p* < 0.001), while there was a weak negative relationship between the overall PRAQ-R2 score and the MOS-SSS score (*r* = −0.024, *p* = 0.672). Additionally, there was a moderate positive relationship between the overall SAI and PRAQ-R2 scores, indicating that higher levels of state anxiety are associated with increased pregnancy-related anxiety.

**Table 5:** Correlation between perceived social support MOS-SSS and scores of the anxiety symptom scales MOS-SSS and SAI

Scale	MOS-SSS		PRAQ-R2		SAI	
	R	P	R	P	R	P
MOS-SSS			− 0.024	0.672	− 0.402^***^	< 0.001
PRAQ-R2	− 0.024	0.672			0.379^***^	< 0.001
SAI	− 0.402^***^	< 0.001	0.379^***^	< 0.001		

*MOS-SSS *Medical Outcomes Study Social Support Scale,* PRAQ-R2 *Pregnancy-Related Anxiety Questionnaire-Revised 2,* SAI *State Anxiety Inventory

*p * *p*-value (^*^ = < 0.05. ^**^ = < 0.01, ^***^ < 0.001), r Spearman correlation result

## Discussion

The findings of this study revealed a high level of perceived social support among Palestinian pregnant women across the three domains of the MOS-SSS, with an average score of 84%. This score indicates a strong presence of emotional, informational, and tangible support. Regarding emotional support, the score was high (265, 86.9%), reflecting significant feelings of being cared for, receiving advice, sharing worries, and experiencing love and affection. The Palestinian population heavily relies on the support of family, relatives, and friends—a cultural value that these results may reflect [49]. The importance of emotional support during pregnancy—such as attentive listening and affectionate interactions—is evident and aligns with findings from previous studies [50, 51]. This emotional support enhances the well-being of expectant mothers and contributes to positive developmental outcomes for their children [52]. Therefore, fostering strong social networks within the community can play a crucial role in promoting healthier pregnancies and nurturing environments.

A significant level of informational support was found in this study, which included advice, guidance, and reassurance from healthcare professionals aimed at alleviating fears and concerns related to pregnancy and childbirth. This support can enhance pregnant women’s access to health-related information, promote feelings of safety, and improve their overall quality of life [53, 54]. Researchers have widely emphasized the value of effective communication between pregnant women and healthcare providers, particularly during the antenatal period [53–55]. Pregnant women may experience increased anxiety, stress, and dissatisfaction due to a lack of informational support from healthcare providers. By addressing both emotional and informational needs, healthcare professionals can enhance maternal well-being and improve overall pregnancy outcomes.

The reported high score of tangible support in this study indicates that Palestinian women gained assistance with daily tasks from husbands, close friends, and relatives, as well as spending quality time together, engaging in activities, and receiving help during illness or discomfort. Previous research has highlighted the tangible and practical aspects of social support in navigating challenging situations and alleviating adversity [56]. It may help women feel loved, cared for, and valued by their families [53, 56]. These findings significantly contribute to the existing body of literature on social relationships among Palestinians. Despite facing prolonged challenges such as occupation, political conflict, and displacement, women can draw strength from their social and familial bonds within extended family networks [57].

The study found no significant relationship between perceived social support and sociodemographic characteristics, such as age, number of previous pregnancies, education, occupation, economic status, or unplanned pregnancy. Conversely, existing literature identifies several predictors of low perceived social support, particularly in low- and middle-income countries (LMICs) like Palestine. These include limited financial resources, poor adherence to treatment, unintended pregnancies, and absence of a supportive partner [16, 58]. A recent systematic review by Bedaso et al. further underscores that financial and economic hardships are major barriers to social support among pregnant women in LMICs, where structural and social challenges often restrict access to essential services and community support networks.

These constraints have the potential to escalate stress levels and lead to social exclusion [16]. However, the high levels of social support reported by pregnant women in this study seem to contradict these findings. The well-documented stressors in the Palestinian context—such as socioeconomic hardships, financial instability, and limited access to healthcare [59]—appear to have little impact on social support.

This study suggests that strong community ties and familial support may play a crucial role in mitigating the negative effects of financial instability. Ultimately, fostering these social networks could be essential for improving mental health outcomes for pregnant women in similar contexts. Future studies should consider contextual and cultural factors when evaluating perceived social support. This approach will help fill gaps in the literature concerning diverse cultural influences [59–61].

In the context of financial instability and socioeconomic hardships faced by Palestinian families, the role of family support becomes increasingly significant. Families often step in to provide economic assistance during challenging times, ensuring that their members have access to essential resources. This support network not only alleviates immediate financial pressures but also develops a sense of security and belonging, which is crucial for mental well-being, particularly for expectant mothers [49, 62].

The mothers participating in this study reported moderate levels of anxiety, as measured by the PRAQ-R2 and SAI scales. These findings align with a previous study conducted in Gaza [63], which found that most pregnant women experienced low to moderate anxiety levels according to the Hamilton Anxiety Rating Scale (HAM-A). Similarly, a study in Saudi Arabia [64] indicated moderate anxiety levels among pregnant women, primarily related to fears surrounding childbirth. Results revealed that nulliparous women exhibited a more pronounced fear of childbirth compared to multiparous women [64]. These results align with the findings of this study, which showed that symptoms of pregnancy-related anxiety significantly decrease among multiparous mothers with more children. However, our findings contrast with a study conducted in Ethiopia [65], which reported a high prevalence of pregnancy-related anxiety linked to factors such as young maternal age, unintended pregnancy, low income, depression, and limited social support. These factors are particularly prevalent in low- and middle-income countries, where socioeconomic instability and gaps in healthcare services often exacerbate maternal stress and vulnerability. It is important to note that discrepancies between studies may arise from variations in measurement tools, study designs, research methodologies, and criteria for sample inclusion and exclusion.

The moderate levels of anxiety reported in this study, even with high social support, may be linked to the unique psychosocial stressors experienced by Palestinian pregnant women. These stressors arise from ongoing political conflict and deteriorating living conditions in Palestine [66]. Such circumstances significantly affect the mental health of pregnant women by limiting their access to healthcare resources and increasing concerns about both their pregnancy and the well-being of their babies [67, 68]. However, there is limited data on the specific effects of political conflict on Palestinian pregnant women’s mental health and their access to maternal healthcare services, which complicates efforts to address these challenges [69].

Moreover, antenatal clinics in Palestine currently lack a structured approach for providing timely, adequate, and appropriate mental health support for pregnant women. Additionally, the healthcare system in Palestine does not employ standardized tools for screening anxiety during pregnancy [70]. Therefore, it is crucial to implement effective health strategies that deliver comprehensive mental health care services, ultimately improving health outcomes and the well-being of pregnant women. These strategies may include training healthcare providers in mental health counselling, establishing referral pathways, and integrating mental health screenings into routine antenatal care.

The current study revealed a significant correlation between antenatal anxiety and employment status. This finding is consistent with previous research [4, 71, 72], which concluded that low economic status often compels pregnant women to remain in the workforce while taking on additional responsibilities and physical demands, potentially heightening their anxiety levels. Challenging working conditions, along with obstetric effects, hormonal fluctuations, and physical discomfort, are well-documented contributors to increased antenatal anxiety [71–73]. A recent systematic review identified further risk factors and potential adverse consequences for working pregnant women, summarizing key occupational hazards affecting this population [74]. These hazards include chemical, psychosocial, physical, ergonomic, and mechanical risks, along with various job-related stressors. Exposure to these risks is associated with serious adverse outcomes, such as low birth weight, preterm birth, miscarriage, hypertension, pre-eclampsia, and a variety of other obstetric complications [74].

On the contrary, several studies have indicated weak or no significant association between employment during pregnancy and the physical or mental health of pregnant women [75, 76], contradicting the findings of the current study. Discrepancies may stem from variations in working conditions, socioeconomic status, and environmental factors across different countries.

In the Palestinian context, increasing restrictions on mobility due to geopolitical conflict and financial constraints may elevate stressors and anxiety levels among employed pregnant women [76]. Additionally, cultural factors in Palestine, similar to those in other Arab countries, play a significant role Women are often viewed as primarily responsible for traditionally feminine tasks and homemaking—such as caring for children, cleaning, and cooking—while men are typically seen as the main breadwinners, responsible for masculine tasks like financial provision and home repairs (49. 53). These unique challenges emphasize the value of developing tailored support systems that address the specific circumstances faced by employed pregnant women. Furthermore, additional research is crucial to explore how these factors interact with employment and influence maternal health outcomes in various contexts [77].

The MOS-SSS score demonstrated a moderate negative correlation with the overall SAI score and a weak negative correlation with the PRAQ-R2 score. These results contrast with findings from a previous systematic review [16], which indicated a strong negative association between social support and anxiety symptoms during pregnancy in seven out of eight studies included in the review. Only one study conducted in Canada [78] reported no significant association. None of the studies utilized the PRAQ-R2 or SAI instruments, which could explain this discrepancy in the sensitivity and specificity of the measurement tools. Additionally, differences in study design, sampling methods, and sociocultural context—particularly cultural norms and the stigma surrounding mental health and social support in Palestine [79]—likely affected the expression and reporting of anxiety symptoms. Future research findings should carefully consider these contextual and cultural factors, which have a major influence on maternal mental health outcomes.

## Study limitations

Causal relationships between anxiety symptoms and perceived social support cannot be established due to the cross-sectional design of this study. The findings should be interpreted with caution, as they represent associations rather than definitive evidence of causality or directionality. Another limitation is that the correlation and regression analyses did not account for potential confounding variables, such as education level, employment status, and number of previous pregnancies. Future research should consider incorporating these factors to yield a more accurate understanding of the observed associations, as their exclusion may have influenced the results. Additionally, variations in participants’ gestational age may have affected data consistency and compromised the uniformity of findings. The reliance on self-reported data also introduces the potential for reporting bias. Moreover, participants’ perceptions of health and anxiety during antenatal visits may have influenced their responses.

## Conclusion

In this study, the pregnant women’s state anxiety was moderately and negatively associated with higher levels of perceived social support. The presence of strong support systems within Palestinian society appears to buffer against anxiety, despite the ongoing political and environmental challenges in the region. Based on these findings, practical efforts should focus on strengthening family- and community-based support mechanisms for pregnant women in Palestine. Policy makers and healthcare providers, particularly midwives and obstetricians, should be trained to routinely assess levels of social support and anxiety during antenatal visits. Integrating brief, culturally sensitive mental health screenings into routine prenatal care could help identify women at risk early. Moreover, targeted interventions—such as support groups, counseling services, and psychosocial education sessions—should be designed to enhance emotional support for expectant mothers, particularly in highly stressful settings.

## Data Availability

No datasets were generated or analysed during the current study.
